# Chronic Effects of Dietary Pesticides on the Gut Microbiome and Neurodevelopment

**DOI:** 10.3389/fmicb.2022.931440

**Published:** 2022-06-30

**Authors:** Jessica Gama, Bianca Neves, Antonio Pereira

**Affiliations:** ^1^Graduate Program in Neuroscience and Cell Biology, Federal University of Pará, Belém, Brazil; ^2^Institute of Chemistry, Federal University of Rio de Janeiro, Rio de Janeiro, Brazil; ^3^Institute of Technology, Federal University of Pará, Belém, Brazil

**Keywords:** microbiome, pesticides, gut- brain axis, neurodevelopment and plasticity, dysbiosis

## Abstract

Many agricultural pesticides include substances that are known to be harmful to human health and while some have been banned from developed countries, they are still being used in developing countries such as Brazil. Recent studies have shown that low-level chronic dietary exposure to pesticides can affect the human gut microbiota. This possible hazardous effect of pesticides on human health has not been specifically recognized by government regulatory agencies. In Brazil, for instance, of the 10 best-selling active ingredients in pesticides in 2019, two are considered extremely toxic, Paraquat and Chlorpyrifos. Even though Paraquat has been banned in Brazil since 2020, the values of maximum residue limits (MRLs) of toxic pesticides allowed in the country are still higher than in other countries. Unfortunately, many developing countries still lack the resources and expertise needed to monitor adequately and systematically the presence of pesticide residues on food. In this work, we raise awareness to the danger the chronic exposure to high dietary levels of pesticides can pose to the public, especially considering their prolonged effects on the gut microbiome.

## Introduction

Agrochemicals or pesticides have been used for centuries in agriculture. Nowadays, they are produced by very competitive industries with synthetic chemical compounds. Since the publication of *Silent Spring* (Carson, 1962), the public has been aware to the deleterious effects of pesticides in the environment and their ultimate impact on human health. The use of pesticides has increased steadily around the world since data began to be recorded and more than 4 million tons are used annually, with China, the United States, and Brazil being the top consumers (FAO, 2022). New research has dramatically charted the damage of pesticides on earth’s more fragile ecosystems, such as the effects of neonicotinoid insecticides (including the “inert” ingredients in their formulations) on the population decline of honeybees and other essential pollinators (Tsvetkov et al., 2017; Straw et al., 2022). A recent study pointed that a healthy gut bacterial community is key to protect honeybees against xenobiotic stressors (Almasri et al., 2022).

After many decades of increasing use of pesticides and the evidence of their residual presence in soil, sediment, and water samples (Estévez et al., 2012), the use of some endocrine-disrupting pesticides, such as Dichlorodiphenyltrichloroethane (DDT), has been abandoned in most countries (though it continues to be used in some countries to control for disease-carrying mosquitoes) (Forbes et al., 2021). Banning a pesticide, however, is usually a long and slow process which is not without significant resistance (Conis, 2022). Furthermore, regulatory agencies are usually slow to catch on the latest scientific findings (Sarkar et al., 2021) and mostly rely on small studies with laboratory animals, which usually do not consider indirect dietary exposure to pesticides *via* long term ingestion of contaminated products.

Though there are many studies on the occupational danger of pesticides, only recently there has emerged a widespread concern with the regular consumption of agricultural products and water laced with pesticides’ residues. New studies have provided ample evidence that pesticides can endanger consumer’s health through microbiome dysbiosis, for instance (Gerage et al., 2017; Mao et al., 2018; Liang et al., 2019; Tsiaoussis et al., 2019; Hu et al., 2021; Zhou and Zhao, 2021). Due to the extensive reciprocal association between the gut microbiome and major homeostatic body networks such as the nervous, endocrine, and immune systems, this has spurred public health concerns and, ideally, should stimulate new guidelines limiting human exposure to pesticides.

## Pesticide Toxicology

Chemical pesticides belong to three major groups: insecticides, herbicides, and fungicides. The evolution of both weed and insect strains displaying resistance to pesticides has worryingly threatened both current and future food security. The chemical industry responded in the 1990s with the development of genetically modified herbicide-resistant crops. Unfortunately, however, this measure ended up leading to an increase in herbicide use (Benbrook, 2012). The arms race between genetically modified seeds and pesticide-resistant invasive species has been tilting toward the latter lately, adding to concerns about collateral environmental damage and their effects on non-target organisms (Owen and Zelaya, 2005). It has encouraged the search for alternative methods of control that are pest-specific, non-harmful to humans, and less likely to promote resistance (Shekhawat et al., 2022).

**FIGURE 1:**
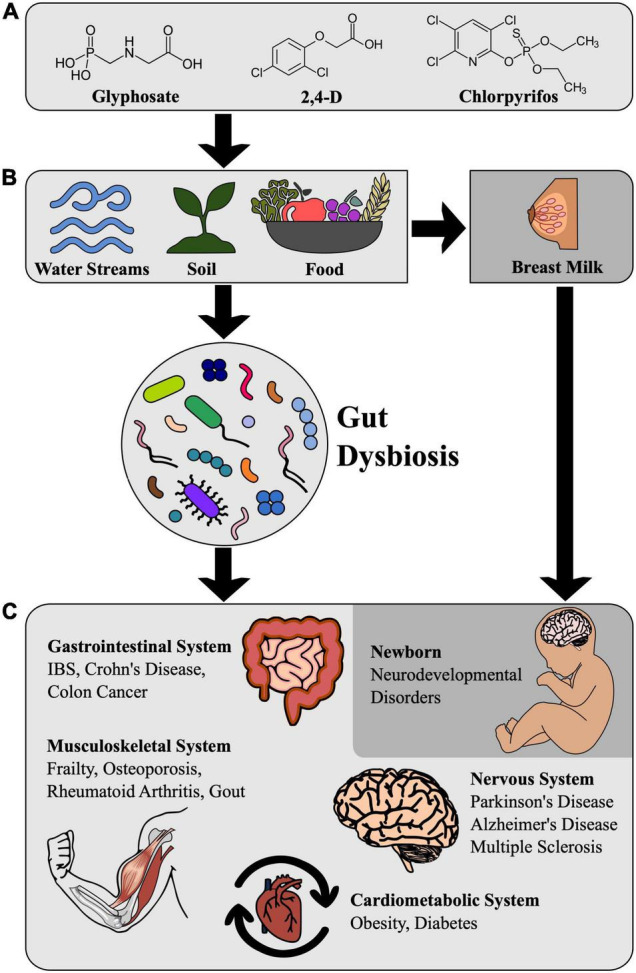
Pesticide-Triggered dysbiosis and impact on human health. **(A)** Structural formulae of widely used pesticides. **(B)** Pesticide residues in soil, water sources, and food can cause gut dysbiosis in humans. Pesticide residues in breast milk can also impair infant neurodevelopment. **(C)** Health consequences of intestinal dysbiosis caused by contamination with pesticide residues.

Aside from occupational exposure, the public is exposed to pesticides mostly by food consumption and drinking water contaminated with pesticide residues (Figure 1A). Fruits and vegetables (FVs) are mainly consumed raw or semi-processed and contain higher residue levels of pesticides when compared to other food groups (Kim et al., 2017). One of the main concerns is that major residual levels (MRLs) of pesticides vary widely among countries (Handford et al., 2015; see Table 1). For instance, a 2016 study (Nakano et al., 2016) found pesticide residues in 42.1% of the orange samples sold to consumers in Brazil. Among the contaminated samples, 3.5% contained residues of bifenthrin and clofentezine above the Brazilian MRL and 12.3% contained unauthorized pesticides (azinphos-ethyl, parathion, myclobutanil, profenofos, and fenitrothion) (Nakano et al., 2016). Another recent study listed the top 10 pesticide residues composing the chronic dietary intake of the Brazilian population: Methyl Bromide, Phosphine, Fipronil, Acephate, Diazinon, Phenine, Terbufos, Diquat, Diurom, Propanil. Methyl bromide is extremely toxic, while Phosphine, Fipronil, Diazinon, Phenine, Terbufod, and Diquat are highly toxic (Marques and da Silva, 2021) (see Table 1 for the list of the three of the most widely used pesticides together with their MRL in food and the possible effects on physiological systems and neurodevelopment).

**TABLE 1:** Widely used pesticides.

	Pesticide	Class	Toxicology class	Environmental hazard potential class	Effects on gut microbiome, nervous system and neurodevelopment	MRL range in food (mg/kg)
1	Glyphosate (C3H8NO5P)	Non-selective herbicide	V—Product Unlikely to Cause Acute Injury	III—Dangerous Product for the Environment	Dechartres et al., 2019 Hu et al., 2021 Leino et al., 2021	0.01–20.0 mg/kg (Brazil)^1^ 1.0–20.0 mg/kg (EU–until 2022)^2^ 0.1–30.0 mg/kg (United States)^3^
2	2,4-dichlorophenoxyacetic acid (C8H6Cl2O3)	Selective herbicide, systemic and post-emergence	IV—Low Toxic Product	III—Dangerous Product for the Environment	Tu et al., 2019	0.01–0.2 mg/kg (Brazil)^1^ 0.05–2 mg/kg (EU)^2^ 0.05 mg/kg—3 mg/kg (United States)^3^
3	Chlorpyrifos (C9H11Cl3NO3PS)	Acaricide and Insecticide for contact and ingestion	I—Extremely Toxic	II—Very Dangerous to the Environment	Guardia-Escote et al., 2020 Perez-Fernandez et al., 2020	0.01–2.0 mg/kg (Brazil)^1^ Banned in The EU^2^ 0.01–2.0 mg/kg United States)^3^

*^1^National Health Surveillance Agency.*

*^2^European Food Safety Authority.*

*^3^United States Environmental Protection Agency.*

## The Gut Microbiota

The human gastrointestinal (GI) tract is one of the largest surfaces regulating and mediating our interaction with the environment. External microorganisms and antigens (xenobiotics) come into close contact with human’s immune and nervous systems through the GI tract. The GI tract is colonized by many microorganisms (Bacteria, Archaea and Eukarya), collectively termed the microbiome. The human gut-microbiota consortium has co-evolved with its host organism to form an intricate and mutually beneficial relationship (Santos et al., 2019).

The human intestinal microbiota is dominated by five phyla: Firmicutes, Bacteroidetes, Actinobacteria, Proteobacteria, and Verrucomicrobia. In adults, more than 80% of the species belong to just two phyla, Firmicutes and Bacteroidetes (Rios-Covian et al., 2017). The Firmicutes/Bacteroidetes ratio is considered a relevant marker of gut dysbiosis (Magne et al., 2020). One of the main functions of the intestinal microbiota is the modification of ingested molecules into bioactive metabolites, which are small molecules produced as intermediate and/or end products of microbial metabolism. Microbial metabolites influence the maturation of the host’s nervous and immune systems (Santos et al., 2019; Zheng et al., 2020) and have a lasting influence on immune and brain homeostasis, host energy metabolism, and maintenance of mucosal integrity.

Xenobiotic-induced changes in the gastrointestinal microbiota can affect the development of the immune system and thus influence the risk of chronic immune mediated conditions, such as inflammatory bowel disease (IBD) (Altajar and Moss, 2020). IBD, namely ulcerative colitis (UC) and Crohn’s disease (CD), has become a global concern and its incidence is rapidly increasing, particularly in less-industrialized countries (Ng et al., 2017; Alatab et al., 2020). In Brazil, for instance, studies demonstrate a remarkable growth in the incidence and estimated prevalence of IBD (Quaresma et al., 2019). For instance, one study showed that the prevalence of Crohn’s disease in Brazil rose from 0.24 (1986–1990) to 24.1 per 100,000 people, while the prevalence of ulcerative colitis increased from 0.99 to 14.1 over the same period of time (Kotze et al., 2019). While this is probable due to a combination of ethiological factors (Ng et al., 2017), the role played by changes in the microbiota caused by chronic exposure to pesticides cannot be overemphasized.

## Pesticides and the Gut Microbiota

When compared to environmental factors, host genetics play only a minor role (<2%) in determining microbiome composition (Rothschild et al., 2018), underscoring the need to include the microbiome as a target in the risk assessment of toxic environmental compounds (Koppel et al., 2017). One of the core functions of the gut microbiota is the modification of xenobiotic and diet-derived molecules into bioactive metabolites (Blumberg and Powrie, 2012; Hooper et al., 2012). Following exposure to xenobiotics, the crosstalk between microbial and human metabolites underpins the xenobiotics’ effects on bacterial physiology and modifications on the overall microbial community (Maurice et al., 2013). Metabolic pathways operating in the human body are thus the result of the combined activities of the human genome and the microbiome. More recently, some studies have shown the potential of gut microbiome profiling as a possible biomarker for environmentally relevant exposure to chemical hazards, such as pesticides (Kandel Gambarte and Wolansky, 2022).

2,4-dichlorophenoxyacetic acid (2,4-D) and glyphosate are two of the most used herbicides and are usually mixed in a compound which is very popular in the market due to the wide use of transgenic crops which are resistant to this herbicide. The mixture is also used to desiccate cereal, bean, and seed crops before harvest, thus increasing the risk of being found as residues in food. Occupational-dose exposure of mice to 2,4-D changed the Firmicutes-to-Bacteroidetes ratio to a dysbiotic one and affected the metabolism of urea, amino acids, and carbohydrates (Tu et al., 2019). According to recent studies (Hu et al., 2021; Leino et al., 2021), glyphosate may have a strong impact on bacterial species in the human microbiome as well, since 54% of the human core gut bacterial species are potentially sensitive to it (Leino et al., 2021).

Though Polychlorinated biphenyls (PCBs) have been banned since 1977 for their toxic effects, they are still present in large quantities in the environment. Human exposure to PCBs occurs mainly through ingestion and have been reported to cause gastrointestinal (GI) distress, intestinal dysbiosis, and may increase the risk of neurodevelopmental disorders (NDD), including autism spectrum disorder (ASD) (Mitchell et al., 2012). A recent work in double-mutant (DM) mice expressing two heritable human mutations showed that developmental exposure to PCBs in the maternal diet caused dysbiosis of the gut microbiota and significant mucosal barrier defects in the ileum and colon of juvenile DM mice, which were also more predisposed to develop defects in neurobehavioral development (Rude et al., 2019). Exposure to PCBs during human pregnancy causes variation in the gut bacterial community of the fetus that lasts until mid-childhood, increasing the relative abundance of Bacillalles, Propionibacteriales, and Propionibacteriaceae (Laue et al., 2019). In a mouse model, low-dose, long-term exposure to chlorpyrifos during the preweaning developmental stage led to gut microbiota dysbiosis, altered expression of muscarinic and GABAergic receptors in the striatum and prefrontal cortex, respectively, and impairment of locomotor activity (Perez-Fernandez et al., 2020). The same group investigated if chlorpyrifos (CPF) administered after birth has differential effects in a mouse model with different apolipoprotein E (APOE) genotypes (Guardia-Escote et al., 2020). The results showed that APOE genotype and CPF exposure diversely affected the types of short-chain fatty acids (SCFA) in the brain (Guardia-Escote et al., 2020).

Though the human gut microbiome is first colonized during delivery, the maternal gut microbiome already modulates fetal neurodevelopment through placental transfer, according to experiments performed with mice (Vuong et al., 2020). Breastfeeding is of great importance during the first postnatal months for the assembly of the human gut microbiome (Figure 1B). Breast milk is associated with higher levels of *Bifidobacterium*, a genus thought to play an important role in infant health and the cessation of breastfeeding results in faster maturation of the gut microbiome, as marked by the steep increase in the phylum Firmicutes (Stewart et al., 2018). However, breast milk can also carry pesticide residues: a recent study in Ethiopia detected DDT and its metabolites in 100% of breastmilk samples and showed that the estimated intake of infants at the first month of breastfeeding was above the provisional tolerable daily intake (PTDI) of DDT set by the FAO/WHO (Mekonen et al., 2021). Another study in China showed that some pesticides exceeded the recommended total daily intake (TDI) in breast milk samples (Dong et al., 2022).

Infants are more vulnerable to pesticides than adults due to the possibility of disruption of developmental processes. Postnatal neurodevelopment proceeds through stages called critical periods where brain circuits are most sensitive to environmental influence. One of the best-known mechanisms by which the gut microbiome impacts the host is by providing key metabolites. Both primary and secondary metabolites generated by bacteria, such as SCFAs and metabolites derived from amino acids such as the neurotransmitter γ-aminobutyric acid (GABA), can influence neurodevelopment. SCFAs can modulate the permeability of the blood-brain barrier allowing access of otherwise impermeable molecules to the brain (Silva et al., 2020), such as GABA, which shapes the connectivity of brain circuits (Tang et al., 2021). GABA-producing pathways are actively expressed in *Bacteroides*, *Parabacteroides*, and *Escherichia* species and the relative abundance levels of fecal *Bacteroides* are negatively correlated with the incidence of depression (Strandwitz et al., 2019). Thus, the increased concentration of both substances due to dysbiosis can contribute to the etiology of neurodevelopmental disorders (Figure 1B).

## Metabolic Pathways

The pathways encoding the production of microbial metabolites are frequently grouped in genomic regions known as metabolic gene clusters (MGCs). Microbial metabolites, such as bile acids, short-chain fatty acids (SCFA), and the tryptophan metabolites quinolinic and kynurenic acids are involved in the control of important metabolic, immune, and neuronal functions of the host. Recent results suggest that bile acids produced by gut bacteria inhibit T_*H*_17 cell function, potentially contributing to inflammatory disorders, including IBD (Paik et al., 2022). While bile acids are known as digestive agents for lipids, they also affect brain function during normal physiological and pathological conditions. Bile acids may be synthesized locally in the brain, but most brain bile acids come from the systemic circulation. Alterations in bile acid metabolism have been discovered as potential biomarkers for neurological conditions (Grant and DeMorrow, 2020).

SCFA are synthesized by gut bacteria in the colon from otherwise indigestible fiber-rich diets and play a key role in neuro-immunoendocrine regulation, including an anti-inflammatory effect, ameliorating diseases in animal models of IBD and allergic asthma (Sun et al., 2017). SCFAs influence gut-brain communication and brain function both directly and indirectly, *via* the immune system, promoting neurogenesis, blood-brain barrier (BBB) integrity, glial function, and influencing behavior and cognitive function (Silva et al., 2020).

Tryptophan (Trp) is an essential amino acid produced by some bacteria in the gut and a key neurotransmitter precursor in the CNS. Some microbial neuro-active metabolites derived from tryptophan, such as quinolinic and kynurenic acids, have been shown to influence the gut-brain-axis (Santos et al., 2019). Analysis of microbiome data from patients with neurological diseases and healthy individuals suggests an association of different sets of Trp-metabolizing bacterial pathways with the etiology of those diseases. In the gut, there are three major metabolic pathways leading from Trp to 5-HT, kynurenine (Kyn), and indole derivatives and the genome of many bacterial species are enriched in these tryptophan metabolic pathways (Kaur et al., 2019).

Like PCB, organochlorine pesticides (OCPs) have been banned since the 1970s but are still detected in the environment (Sparling, 2016). Chronic exposure of mice to OCPs impair the intestinal microbiota and modify the hepatic and enteric bile acid profiles with profound influences in host metabolism (Liu et al., 2017). Exposure of mice to CPF led to intestinal inflammation and abnormal intestinal permeability while altering the composition of gut microbiota and urine metabolites related to the metabolism of amino acids, energy, short-chain fatty acids (SCFAs), phenyl derivatives, and bile acids (Zhao et al., 2016).

Other pesticides that have been shown to disturb the metabolism profiles of mice are nitenpyran, imanzalil, penzonazole, and propamocarb (for review, see Zhou and Zhao, 2021). The first is an insecticide and the last three are fungicides. Current studies have confirmed that the exposure to those pesticides can cause the dysbiosis of gut microbiota and metabolic disorders in the host (for review, see Zhou and Zhao, 2021; Figure 1C).

## Neural Effects

Gut-brain modules are gene groups within the microbiome associated with the synthesis of neuroactive metabolites. A recent study proposed the existence of 56 such modules, all structurally centered around a different neuroactive molecule, such as dopamine or serotonin (Valles-Colomer et al., 2019).

Exposure to pesticides and other toxicants is of special concern during the critical periods of development of the central nervous system (Figure 1C). During critical periods, the circuits of the brain are refined and adapted to the specific host’s environment. Dysbiosis during critical periods of development is a key element linking pesticide exposure to autism spectrum disorder (ASD) (He et al., 2022). Autism Spectrum Disorders (ASDs) are a group of developmental disabilities that can cause significant social, communication and behavioral deficits which affect approximately 1 in 44 children in the United States (Maenner et al., 2021). The prevalence rates of ASD are increasing worldwide and while there’s no way to pinpoint an exact reason for this increase, it’s likely that environmental factors are also responsible for this rise in ASD and other developmental disorders (Bennett et al., 2022).

Regarding the effects of long-term dietary exposure to pesticides on the incidence of neurodegenerative disorders, such as Alzheimer’s and Parkinson’s disease, there is increasing evidence that the steady exposure of the gut-microbiome to toxicants and the resulting dysbiosis can trigger a cascade of events that can cause these disorders in the long-term (Santos et al., 2019; Figure 1C). For instance, α-syn aggregates initiated in the enteric nervous system may be transmitted in a prion-like manner to the CNS through the VN and seed the Lewy body inclusions seen in the substantia nigra of Parkinson’s patients (Santos et al., 2019).

## Discussion

Outright banning of pesticide use is not currently feasible due to food security concerns and the lack of viable alternatives for large-scale replacement. A case in point is the expiration of current approval for glyphosate use in the European Union planned for December 2022 and the potential for a glyphosate ban (Kudsk and Mathiassen, 2020). Though not considered a carcinogenic agent by the EPA (2017), glyphosate may be nonetheless capable of causing dysbiosis in the gut microbiome and indirectly affecting human health (see above). Glyphosate is widely used around the world and without it many farmers expect an increase in herbicide-resistant biotypes and a consequent decrease in productivity. Besides, there are legitimate concerns regarding the loss of benefits of using glyphosates for weed control in environmental restoration projects and the possibility that land care programs (no-tillage/conservation) agriculture, which are widely used in Brazil, one of the largest agricultural producers in the world and viewed as an effective strategy to prevent soil erosion and loss of nutrients, would be unpractical without glyphosate.

Biopesticides and genetically modified organisms (GMO) are a promising alternative to reduce/replace the use of agrochemicals. Biopesticides include microorganisms that target specific pathogens, biochemical pesticides that control insect behavior, and plant-incorporated protectants (Baranski et al., 2019; Arif et al., 2020; Fletcher et al., 2020). However, large-scale utilization of biopesticides will depend on dealing with four challenges: cost-effectiveness, farmers’ awareness, eventual incompatibilities between pesticides and microbial inoculants, and safety to non-target organisms (including humans). The most common genetically engineered insect resistant crop is based on proteins encoded by genes derived from the bacterium *Bacillus thuringiensis* (Bt) which are not toxic to humans or non-target wildlife (Duan et al., 2008; Raymond and Federici, 2017). Other strategies, such as RNA-based biopesticides, seem to present minimal risks to humans and any unintended impacts to the environment are expected to be most apparent in species closely related to the target (Fletcher et al., 2020). We expect that promoting research on synthetic biology in applied ecology, micro-organisms, and plant engineering will offer new possibilities for the future of agriculture.

Equally important is to promote international cooperation to support the monitoring capabilities of developing countries, which usually lack the expertise and resources to implement effective monitoring programs for pesticide contaminants in both drinking water and food (Sarkar et al., 2021).

## Author Contributions

JG, BN, and AP wrote the manuscript. All authors contributed to the article and approved the submitted version.

## Conflict of Interest

The authors declare that the research was conducted in the absence of any commercial or financial relationships that could be construed as a potential conflict of interest.

## Publisher’s Note

All claims expressed in this article are solely those of the authors and do not necessarily represent those of their affiliated organizations, or those of the publisher, the editors and the reviewers. Any product that may be evaluated in this article, or claim that may be made by its manufacturer, is not guaranteed or endorsed by the publisher.

## References

[B1] Alatab S. Sepanlou S. G. Ikuta K. Vahedi H. Bisignano C. Safiri S. (2020). The global, regional, and national burden of inflammatory bowel disease in 195 countries and territories, 1990–2017: a systematic analysis for the Global Burden of Disease Study 2017. *Lancet Gastroenterol. Hepatol.* 5 17–30. 10.1016/S2468-1253(19)30333-4 31648971 PMC7026709

[B2] Almasri H. Liberti J. Brunet J.-L. Engel P. Belzunces L. P. (2022). Mild chronic exposure to pesticides alters physiological markers of honey bee health without perturbing the core gut microbiota. *Sci. Rep.* 12:4281. 10.1038/s41598-022-08009-2 35277551 PMC8917129

[B3] Altajar S. Moss A. (2020). Inflammatory Bowel Disease Environmental Risk Factors: diet and Gut Microbiota. *Curr. Gastroenterol. Rep.* 22:57. 10.1007/s11894-020-00794-y 33044636

[B4] Arif I. Batool M. Schenk P. M. (2020). Plant Microbiome Engineering: expected Benefits for Improved Crop Growth and Resilience. *Trends Biotechnol.* 38 1385–1396. 10.1016/j.tibtech.2020.04.015 32451122

[B5] Baranski R. Klimek-Chodacka M. Lukasiewicz A. (2019). Approved genetically modified (GM) horticultural plants: a 25-year perspective. *Folia Hortic.* 31 3–49. 10.2478/fhort-2019-0001

[B6] Benbrook C. M. (2012). Impacts of genetically engineered crops on pesticide use in the U.S. – the first sixteen years. *Environ. Sci. Eur.* 24:24. 10.1186/2190-4715-24-24

[B7] Bennett D. H. Busgang S. A. Kannan K. Parsons P. J. Takazawa M. Palmer C. D. (2022). Environmental exposures to pesticides, phthalates, phenols and trace elements are associated with neurodevelopment in the CHARGE study. *Environ. Int.* 161:107075. 10.1016/j.envint.2021.107075 35085933 PMC9317896

[B8] Blumberg R. Powrie F. (2012). Microbiota, disease, and back to health: a metastable journey. *Sci. Transl. Med.* 4:137rv7. 10.1126/scitranslmed.3004184 22674557 PMC5020897

[B9] Carson R. (1962). *Silent spring.* Boston: Houghton Mifflin.

[B10] Conis E. (2022). *How to sell a poison: the rise, fall, and toxic return of DDT*. First edition. New York: Bold Type Books.

[B11] Dechartres J. Pawluski J. L. Gueguen M. M. Jablaoui A. Maguin E. Rhimi M. (2019). Glyphosate and glyphosate-based herbicide exposure during the peripartum period affects maternal brain plasticity, maternal behaviour and microbiome. *J. Neuroendocrinol.* 31:e12731. 10.1111/jne.12731 31066122

[B12] Dong Y. Yin S. Zhang J. Guo F. Aamir M. Liu S. (2022). Exposure patterns, chemical structural signatures, and health risks of pesticides in breast milk: a multicenter study in China. *Sci. Total Environ.* 830:154617. 10.1016/j.scitotenv.2022.154617 35307419

[B13] Duan J. J. Marvier M. Huesing J. Dively G. Huang Z. Y. (2008). A Meta-Analysis of Effects of Bt Crops on Honey Bees (Hymenoptera: apidae). *PLoS One* 3:e1415. 10.1371/journal.pone.0001415 18183296 PMC2169303

[B14] EPA (2017). *Revised Glyphosate Issue Paper: Evaluation of Carcinogenic Potential.* Washington, D.C: Environmental Protection Agency.

[B15] Estévez E. Cabrera M. del C. Molina-Díaz A. Robles-Molina J. Palacios-Díaz M. (2012). Screening of emerging contaminants and priority substances (2008/105/EC) in reclaimed water for irrigation and groundwater in a volcanic aquifer (Gran Canaria. *Canary Islands, Spain)*. *Sci. Total Environ.* 433 538–546. 10.1016/j.scitotenv.2012.06.031 22858460

[B16] FAO (2022). *FAOSTAT. Food Agric. Data.* Available Online at: https://www.fao.org/faostat/en/#home (Accessed May 24, 2022).

[B17] Fletcher S. J. Reeves P. T. Hoang B. T. Mitter N. (2020). A Perspective on RNAi-Based Biopesticides. *Front. Plant Sci.* 11:51. 10.3389/fpls.2020.00051 32117388 PMC7028687

[B18] Forbes P. Naudé Y. Strumpher J. (2021). “Ongoing Use and Monitoring of DDT in South Africa,” in *Persistent organic pollutants in the environment: origin and role*, eds Kumar N. Shukla V. (Boca Raton: CRC Press), 378.

[B19] Gerage J. M. Meira A. P. G. da Silva M. V. (2017). Food and nutrition security: pesticide residues in food. *Nutrire* 42:3. 10.1186/s41110-016-0028-4

[B20] Grant S. M. DeMorrow S. (2020). Bile Acid Signaling in Neurodegenerative and Neurological Disorders. *Int. J. Mol. Sci.* 21:5982. 10.3390/ijms21175982 32825239 PMC7503576

[B21] Guardia-Escote L. Basaure P. Biosca-Brull J. Cabré M. Blanco J. Pérez-Fernández C. (2020). APOE genotype and postnatal chlorpyrifos exposure modulate gut microbiota and cerebral short-chain fatty acids in preweaning mice. *Food Chem. Toxicol.* 135:110872. 10.1016/j.fct.2019.110872 31622728

[B22] Handford C. E. Elliott C. T. Campbell K. (2015). A review of the global pesticide legislation and the scale of challenge in reaching the global harmonization of food safety standards: global Harmonization of Pesticide Legislation. *Integr. Environ. Assess. Manag.* 11 525–536. 10.1002/ieam.1635 25765969

[B23] He X. Tu Y. Song Y. Yang G. You M. (2022). The relationship between pesticide exposure during critical neurodevelopment and autism spectrum disorder: a narrative review. *Environ. Res.* 203:111902. 10.1016/j.envres.2021.111902 34416252

[B24] Hooper L. V. Littman D. R. Macpherson A. J. (2012) Interactions between the microbiota and the immune system. *Science* 336, 1268–1273. 10.1126/science.1223490 22674334 PMC4420145

[B25] Hu J. Lesseur C. Miao Y. Manservisi F. Panzacchi S. Mandrioli D. (2021). Low-dose exposure of glyphosate-based herbicides disrupt the urine metabolome and its interaction with gut microbiota. *Sci. Rep.* 11:3265. 10.1038/s41598-021-82552-2 33547360 PMC7864973

[B26] Kandel Gambarte P. C. Wolansky M. J. (2022). The gut microbiota as a biomarker for realistic exposures to pesticides: a critical consideration. *Neurotoxicol. Teratol.* 91:107074. 10.1016/j.ntt.2022.107074 35063647

[B27] Kaur H. Bose C. Mande S. S. (2019). Tryptophan Metabolism by Gut Microbiome and Gut-Brain-Axis: an in silico Analysis. *Front. Neurosci.* 13:1365. 10.3389/fnins.2019.01365 31920519 PMC6930238

[B28] Kim K.-H. Kabir E. Jahan S. A. (2017). Exposure to pesticides and the associated human health effects. *Sci. Total Environ.* 575 525–535. 10.1016/j.scitotenv.2016.09.009 27614863

[B29] Koppel N. Maini Rekdal V. Balskus E. P. (2017). Chemical transformation of xenobiotics by the human gut microbiota. *Science* 356 eaag2770. 10.1126/science.aag2770 28642381 PMC5534341

[B30] Kotze P. G. Underwood F. Damiao A. O. Ferraz J. Saad-Hossne R. Toro M. (2019). P105 THE PROGRESSION OF INFLAMMATORY BOWEL DISEASE THROUGHOUT LATIN AMERICA: a SYSTEMATIC REVIEW. *Inflamm. Bowel Dis.* 25 S51–S51. 10.1093/ibd/izy393.113

[B31] Kudsk P. Mathiassen S. K. (2020). Pesticide regulation in the European Union and the glyphosate controversy. *Weed Sci.* 68 214–222. 10.1017/wsc.2019.59

[B32] Laue H. E. Brennan K. J. M. Gillet V. Abdelouahab N. Coull B. A. Weisskopf M. G. (2019). Associations of prenatal exposure to polybrominated diphenyl ethers and polychlorinated biphenyls with long-term gut microbiome structure: a pilot study. *Environ. Epidemiol.* 3:e039. 10.1097/EE9.0000000000000039 30778401 PMC6376400

[B33] Leino L. Tall T. Helander M. Saloniemi I. Saikkonen K. Ruuskanen S. (2021). Classification of the glyphosate target enzyme (5-enolpyruvylshikimate-3-phosphate synthase) for assessing sensitivity of organisms to the herbicide. *J. Hazard. Mater.* 408:124556. 10.1016/j.jhazmat.2020.124556 33243645

[B34] Liang Y. Zhan J. Liu D. Luo M. Han J. Liu X. (2019). Organophosphorus pesticide chlorpyrifos intake promotes obesity and insulin resistance through impacting gut and gut microbiota. *Microbiome* 7:19. 10.1186/s40168-019-0635-4 30744700 PMC6371608

[B35] Liu Q. Shao W. Zhang C. Xu C. Wang Q. Liu H. (2017). Organochloride pesticides modulated gut microbiota and influenced bile acid metabolism in mice. *Environ. Pollut.* 226 268–276. 10.1016/j.envpol.2017.03.068 28392238

[B36] Maenner M. J. Shaw K. A. Bakian A. V. Bilder D. A. Durkin M. S. Esler A. (2021). Prevalence and Characteristics of Autism Spectrum Disorder Among Children Aged 8 Years — Autism and Developmental Disabilities Monitoring Network, 11 Sites. United States, 2018. *MMWR Surveill. Summ.* 70 1–16. 10.15585/mmwr.ss7011a1 34855725 PMC8639024

[B37] Magne F. Gotteland M. Gauthier L. Zazueta A. Pesoa S. Navarrete P. (2020). The Firmicutes/Bacteroidetes Ratio: a Relevant Marker of Gut Dysbiosis in Obese Patients? *Nutrients* 12:1474. 10.3390/nu12051474 32438689 PMC7285218

[B38] Mao Q. Manservisi F. Panzacchi S. Mandrioli D. Menghetti I. Vornoli A. (2018). The Ramazzini Institute 13-week pilot study on glyphosate and Roundup administered at human-equivalent dose to Sprague Dawley rats: effects on the microbiome. *Environ. Health* 17:50. 10.1186/s12940-018-0394-x 29843725 PMC5972442

[B39] Marques J. M. G. da Silva M. V. (2021). Estimativa de ingestão crônica de resíduos de agrotóxicos por meio da dieta. *Rev. Saúde Pública* 55:36. 10.11606/s1518-8787.2021055002197 34190889 PMC8225320

[B40] Maurice C. F. Haiser H. J. Turnbaugh P. J. (2013). Xenobiotics shape the physiology and gene expression of the active human gut microbiome. *Cell* 152, 39–50. 10.1016/j.cell.2012.10.052 23332745 PMC3552296

[B41] Mekonen S. Ambelu A. Wondafrash M. Kolsteren P. Spanoghe P. (2021). Exposure of infants to organochlorine pesticides from breast milk consumption in southwestern Ethiopia. *Sci. Rep.* 11:22053. 10.1038/s41598-021-01656-x 34764390 PMC8585979

[B42] Mitchell M. M. Woods R. Chi L.-H. Schmidt R. J. Pessah I. N. Kostyniak P. J. (2012). Levels of select PCB and PBDE congeners in human postmortem brain reveal possible environmental involvement in 15q11-q13 duplication autism spectrum disorder. *Environ. Mol. Mutagen.* 53 589–598. 10.1002/em.21722 22930557 PMC3739306

[B43] Nakano V. E. Kussumi T. A. Lemes V. R. R. Kimura I. de A. Rocha S. B. (2016). Evaluation of pesticide residues in oranges from São Paulo, Brazil. *Food Sci. Technol.* 36 40–48. 10.1590/1678-457X.6837

[B44] Ng S. C. Shi H. Y. Hamidi N. Underwood F. E. Tang W. Benchimol E. I. (2017). Worldwide incidence and prevalence of inflammatory bowel disease in the 21st century: a systematic review of population-based studies. *The Lancet* 390 2769–2778. 10.1016/S0140-6736(17)32448-0 29050646

[B45] Owen M. D. Zelaya I. A. (2005). Herbicide-resistant crops and weed resistance to herbicides. *Pest Manag. Sci.* 61 301–311. 10.1002/ps.1015 15668920

[B46] Paik D. Yao L. Zhang Y. Bae S. D’Agostino G. D. Zhang M. (2022). Human gut bacteria produce TH 17-modulating bile acid metabolites. *Nature* 603 907–912. 10.1038/s41586-022-04480-z 35296854 PMC9132548

[B47] Perez-Fernandez C. Morales-Navas M. Guardia-Escote L. Garrido-Cárdenas J. A. Colomina M. T. Giménez E. (2020). Long-term effects of low doses of Chlorpyrifos exposure at the preweaning developmental stage: a locomotor, pharmacological, brain gene expression and gut microbiome analysis. *Food Chem. Toxicol.* 135:110865. 10.1016/j.fct.2019.110865 31618664

[B48] Quaresma A. B. Kaplan G. G. Kotze P. G. (2019). The globalization of inflammatory bowel disease: the incidence and prevalence of inflammatory bowel disease in Brazil. *Curr. Opin. Gastroenterol.* 35 259–264. 10.1097/MOG.0000000000000534 30973356

[B49] Raymond B. Federici B. A. (2017). In defence of Bacillus thuringiensis, the safest and most successful microbial insecticide available to humanity—a response to EFSA. *FEMS Microbiol. Ecol.* 93:fix084. 10.1093/femsec/fix084 28645183 PMC5812528

[B50] Rios-Covian D. Salazar N. Gueimonde M. de los Reyes-Gavilan C. G. (2017). Shaping the Metabolism of Intestinal *Bacteroides* Population through Diet to Improve Human Health. *Front. Microbiol.* 8:376. 10.3389/fmicb.2017.00376 28326076 PMC5339271

[B51] Rothschild D. Weissbrod O. Barkan E. Kurilshikov A. Korem T. Zeevi D. (2018). Environment dominates over host genetics in shaping human gut microbiota. *Nature* 555 210–215. 10.1038/nature25973 29489753

[B52] Rude K. M. Pusceddu M. M. Keogh C. E. Sladek J. A. Rabasa G. Miller E. N. (2019). Developmental exposure to polychlorinated biphenyls (PCBs) in the maternal diet causes host-microbe defects in weanling offspring mice. *Environ. Pollut.* 253 708–721. 10.1016/j.envpol.2019.07.066 31336350 PMC6719698

[B53] Santos S. F. de Oliveira H. L. Yamada E. S. Neves B. C. Pereira A. (2019). The Gut and Parkinson’s Disease—A Bidirectional Pathway. *Front. Neurol.* 10:574. 10.3389/fneur.2019.00574 31214110 PMC6558190

[B54] Sarkar S. Dias Bernardes Gil J. Keeley J. Möhring N. Jansen K. (2021). *The Use Of Pesticides In Developing Countries And Their Impact On Health And The Right To Food.* Strasbourg: European Parliament.

[B55] Shekhawat K. Rathore S. S. Babu S. Raj R. Chauhan B. S. (2022). Exploring alternatives for assessing and improving herbicide use in intensive agroecosystems of South Asia: a review. *Adv. Weed Sci.* 40 e0202200116. 10.51694/AdvWeedSci/2022;40:seventy-five005

[B56] Silva Y. P. Bernardi A. Frozza R. L. (2020). The Role of Short-Chain Fatty Acids From Gut Microbiota in Gut-Brain Communication. *Front. Endocrinol.* 11:25. 10.3389/fendo.2020.00025 32082260 PMC7005631

[B57] Sparling D. W. (2016). “Chapter 4 – organochlorine pesticides,” in *Ecotoxicology Essentials*, ed. Sparling D. W. (Cambridge, MA: Academic Press), 69–107. 10.1016/B978-0-12-801947-4.00004

[B58] Stewart C. J. Ajami N. J. O’Brien J. L. Hutchinson D. S. Smith D. P. Wong M. C. (2018). Temporal development of the gut microbiome in early childhood from the TEDDY study. *Nature* 562 583–588. 10.1038/s41586-018-0617-x 30356187 PMC6415775

[B59] Strandwitz P. Kim K. H. Terekhova D. Liu J. K. Sharma A. Levering J. (2019). GABA-modulating bacteria of the human gut microbiota. *Nat. Microbiol.* 4:396. 10.1038/s41564-018-0307-3 30531975 PMC6384127

[B60] Straw E. A. Thompson L. J. Leadbeater E. Brown M. J. F. (2022). ‘Inert’ ingredients are understudied, potentially dangerous to bees and deserve more research attention. *Proc. R. Soc. B Biol. Sci.* 289:20212353. 10.1098/rspb.2021.2353 35232234 PMC8889201

[B61] Sun M. Wu W. Liu Z. Cong Y. (2017). Microbiota metabolite short chain fatty acids, GPCR, and inflammatory bowel diseases. *J. Gastroenterol.* 52 1–8. 10.1007/s00535-016-1242-9 27448578 PMC5215992

[B62] Tang X. Jaenisch R. Sur M. (2021). The role of GABAergic signalling in neurodevelopmental disorders. *Nat. Rev. Neurosci.* 22 290–307. 10.1038/s41583-021-00443-x 33772226 PMC9001156

[B63] Tsiaoussis J. Antoniou M. N. Koliarakis I. Mesnage R. Vardavas C. I. Izotov B. N. (2019). Effects of single and combined toxic exposures on the gut microbiome: current knowledge and future directions. *Toxicol. Lett.* 312 72–97. 10.1016/j.toxlet.2019.04.014 31034867

[B64] Tsvetkov N. Samson-Robert O. Sood K. Patel H. S. Malena D. A. Gajiwala P. H. (2017). Chronic exposure to neonicotinoids reduces honey bee health near corn crops. *Science* 356 1395–1397. 10.1126/science.aam7470 28663503

[B65] Tu P. Gao B. Chi L. Lai Y. Bian X. Ru H. (2019). Subchronic low-dose 2,4-D exposure changed plasma acylcarnitine levels and induced gut microbiome perturbations in mice. *Sci. Rep.* 9:4363. 10.1038/s41598-019-40776-3 30867497 PMC6416245

[B66] Valles-Colomer M. Falony G. Darzi Y. Tigchelaar E. F. Wang J. Tito R. Y. (2019). The neuroactive potential of the human gut microbiota in quality of life and depression. *Nat. Microbiol.* 4 623–632. 10.1038/s41564-018-0337-x 30718848

[B67] Vuong H. E. Pronovost G. N. Williams D. W. Coley E. J. L. Siegler E. L. Qiu A. (2020). The maternal microbiome modulates fetal neurodevelopment in mice. *Nature* 586 281–286. 10.1038/s41586-020-2745-3 32968276 PMC7554197

[B68] Zhao Y. Zhang Y. Wang G. Han R. Xie X. (2016). Effects of chlorpyrifos on the gut microbiome and urine metabolome in mouse (Mus musculus). *Chemosphere* 153 287–293. 10.1016/j.chemosphere.2016.03.055 27018521

[B69] Zheng D. Liwinski T. Elinav E. (2020). Interaction between microbiota and immunity in health and disease. *Cell Res.* 30 492–506. 10.1038/s41422-020-0332-7 32433595 PMC7264227

[B70] Zhou M. Zhao J. (2021). A Review on the Health Effects of Pesticides Based on Host Gut Microbiome and Metabolomics. *Front. Mol. Biosci.* 8:632955. 10.3389/fmolb.2021.632955 33628766 PMC7897673

